# Prognostic value of bone scan index as an imaging biomarker in metastatic prostate cancer: a meta-analysis

**DOI:** 10.18632/oncotarget.19680

**Published:** 2017-07-29

**Authors:** Dongyang Li, Hang Lv, Xuanyu Hao, Yudi Dong, Huixu Dai, Yongsheng Song

**Affiliations:** ^1^ Department of Urology, Shengjing Hospital of China Medical University, Shenyang, Liaoning 110004, P.R. China; ^2^ Department of Urology, Liaoning Cancer Hospital and Institute, Cancer Hospital of China Medical University, Shenyang, Liaoning 110042, P.R. China; ^3^ Department of Rheumatology and Immunology, Shengjing Hospital of China Medical University, Shenyang, Liaoning 110022, P.R. China; ^4^ Department of Medical Research Center, Shengjing Hospital of China Medical University, Shenyang, Liaoning 110004, P.R. China; ^5^ Department of Clinical Epidemiology and Evidence-based Medicine, The First Hospital of China Medical University, Shenyang, Liaoning 110001, P.R. China

**Keywords:** metastatic prostate cancer, bone scan index, prognosis, survival, meta-analysis

## Abstract

**Background:**

The prognostic value of the bone scan index (BSI) in metastatic prostate cancer (mPCa) remained controversial. Therefore, we performed a meta-analysis to determine the predictive value of BSI and survival in patients with mPCa.

**Materials and Methods:**

A literature search was performed in PubMed, Embase, Web of Science and Cochrane library databases. Hazard ratios (HRs), concordance indices (C-indices) were extracted to estimate the relationship between BSI and survival in patients with mPCa. Subgroup analyses were conducted on different types of mPCa, ethnics, cut-off values and sample sizes.

**Results:**

14 high quality studies involving 1295 patients with mPCa were included in this meta-analysis. The pooled results indicated that high basline BSI and elevated BSI change on treatment (ΔBSI) were significantly predictive of poor overall survial (HR = 1.29, *P* < 0.001; HR = 1.27, *P* < 0.001, respectively). Baseline BSI was also significantly related to cancer specific survival (HR = 1.65, *P* = 0.019) and prostate specific antigen recurrence survival (HR = 2.26, *P* < 0.001). Subgroup analysis supported main results. Moreover, BSI could increase the C-indices of predictive models.

**Conclusions:**

Baseline BSI and ΔBSI may be beneficial to mPCa prognosis in clinical monitor and treatment. Further high quality studies with larger sample size are required in the future.

## INTRODUCTION

Prostate cancer (PCa) has been the first common malignancy bothering western men [1]. Among all the PCa-related death, over 85% patients died from bone metastasis [2]. Currently, the standard first-line treatment of metastatic PCa (mPCa) is androgen deprivation therapy (ADT). Some patients with indolent PCa may survive for decades, however, most patients eventually become resistant to ADT and develop to castration-resistant PCa (CRPC). New bone metastases usually occur in CRPC patients, which indicates a high risk of poor outcome. Besides, CRPC patients need second-line treatment such as abiraterone, chemotherapy and bone-targeted radiotherapy [3]. Some pathological and biochemical tests involved in making prognosis [4], but in fact, no predictors are precise enough for the clinical practice. There is an urgent need for new effective indicators for risk stratification and predicting outcome on treatment decision.

Bone scintigraphy is a widely used examination for patients with mPCa to access metastatic disease burden or treatment effects. However, bone scintigraphy images only provide intensity and size of osseous lesions, which may lead to inaccurate subjective evaluation. To mitigate the shortcoming of bone scintigraphy, Massimo Imbriaco first reported a quantifiable and objective method, bone scan index (BSI), in 1998 [5]. BSI represents the percentage of bone weight affected by tumor to the entire skeleton mass. Initially, the BSI manual calculation was time-consuming and required experienced readers, so it was not introduced into the clinical application. Recently, an automated software package to calculate BSI was commercially available [6–7]. To date, the prognosis ability of BSI in patients with mPCa has been discussed in several studies, but a few of these studies draw controversial conclusions [15, 17]. The aim of this present study was to use a meta-analysis to quantitatively and comprehensively summarize the evidence on the prognostic performance of BSI in patients with mPCa.

## RESULTS

### Study search and characteristics

**Figure 1:**
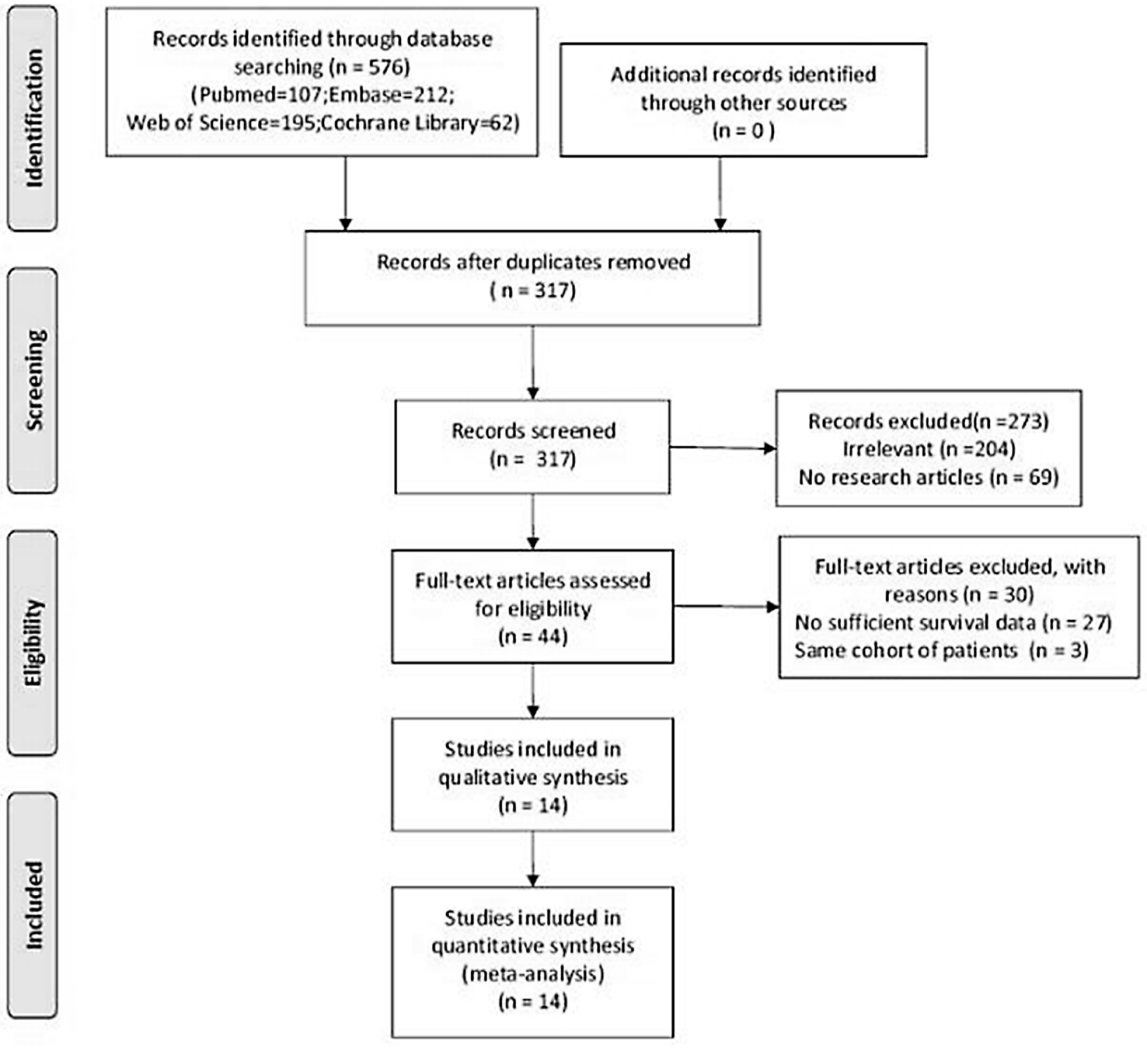
Flow chart of literature search and study selection

The process of literature selection was shown in a flow diagram (Figure 1). A total of 576 studies were initially identified with the keywords used to search the databases. By screening the titles and abstracts, we retrieved 44 potential studies. 30 studies were then excluded after further fully reviewed because they were insufficient of data (27 studies) or consist of same patients (3 studies). Though some studies were in the same institute, the sample patients were at different stage and received different treatment, so we regarded them as different cohorts [10, 16, 21]. Finally, 14 cohort studies met the inclusion criteria for our meta-analysis.

The baseline characteristics of the studies were shown in Table 1. The articles were published from 2010 to 2017, including 1295 patients. Among them, 11 studies [11–21] used same model of BSI software, EXINI bone (EXINI Diagnostics AB, Lund, Sweden), while the other 3 studies [8–10] used BONENAVI system (Fujifilm RI Pharma, Co. Ltd., Tokyo, Japan). Overall, the whole 14 studies reported the prognostic ability of BSI in the survival of patients with mPCa.

**Table 1:** Baseline characteristics of included studies

Study ID	Country	Duratioin	Sample size	Median age (years)	Mean serum PSA(ng/ml)	Mean Gleason score	Follow up (months)	Type of PCa	Treatment	HR	95% CI	Cut-off value	Survival outcome	Multivariate analysis	Study quality (NOS score)
Mitsui et al. 2012 [8]	Japan	2004–2011	42	73 (52–86)	65.3 (0.1–3584.1)	8	70	mCRPC	chemotherapy	3.87	1.24–12.14	3	OS	yes	7
										2.67	1.211– 5.886	∆BSI			
Miyoshi et al. 2016 [9]	Japan	2010–2014	60	72 (55–89)	247.0 (9.7–4206.0)	8.53	57.8	hormone-naive PCa	ADT/ chemotherapy	4.676	1.238–17.661	1.90	OS	yes	7
Anand et al. 2016 [11]	Sweden	2012–2014	80	71 (54–84)	46 (3.7–4625)	NR	40.8	mCRPC	ENZ	NR	NR	∆BSI NR	OS	no	6
Reza et al. 2014 [12]	Sweden	1996–2010	121	71 (65–77)	72 (20–187)	8	60	mPCa	ADT	1.26	1.16–1.37	1	OS	yes	7
										1.19	1.09–1.29	∆BSI			
Armstrong et al. 2014 [13]	USA	2010–2012	85	75	NR	NR	37	mCRPC	tasquinimod	1.64	1.22–2.21	1	OS	yes	7
										1.58	1.04–2.39	∆BSI			
										2.14	NR	∆BSI	PFS		
Lindgren et al. 2017 [14]	Sweden	2009–2012	48	73 (53–92)	84 (4–5740)	8	60	NR	NR	1.26	1.13–1.41	0.39	OS	no	6
Meirelles et al. 2010 [15]	USA	1997–2000	43	68 (47–86)	NR	NR	60	mPCa & mCRPC	NR	1.54	0.63–3.74	1.27	OS	yes	7
Reza et al. 2016(1) [16]	Sweden	2011–2014	104	71 (66–75)	77 (20–180)	NR	36	mCRPC	AA	1.1	1.009–1.232	∆BSI	OS	yes	7
Alva et al. 2017 [17]	USA	2013–2014	65	71.8 (46.1–92.2)	NR	8	18	mCRPC	radium-223	1.01	0.85–1.20	5	OS	no	6
Miyoshi et al. 2016 (2) [10]	Japan	2012–2016	40	75.5 (56.7–86.5)	26.4 (1.8–4645.0)	NR	36	mCRPC	AA/ENZ	1.20	0.31–4.70	1	OS	Yes	7
										8.97	1.65–48.79	∆BSI			
Poulsen et al. 2015 [18]	Denmark	until 2013	88	72 (52–92)	73 (4–5740)	7.7	49	hormone-sensitive mPCa	ADT	1.34	1.07–1.67	1	CSS	yes	7
Ulmert et al. 2012 [19]	USA	until 2006	384	69 (65–73)	16.8 (8.5–49.4)	7	120	PCa & mCRPC	NR	2.055	1.572–2.687	1	CSS	no	6
Dennis et al. 2012 [20]	USA	1997–2005	88	68 (44–83)	95.95 (0.52–2282.15)	8	6	mCRPC	chemotherapy	2.226	1.716–2.736	∆BSI	PSA-RS	yes	7
Reza et al. 2016 (2) [21]	Sweden	2011–2013	47	68 (50–82)	83.1 (4–1294)	NR	30	mCRPC	ODM-201	4.27	0.84–21.6	1	PSA-RS	no	6
										2.66	1.03–6.84	∆BSI	PFS		

NR: not reported ; ENZ: Enzalutamide ; AA: Abiraterone Acetate; ADT: Androgen deprivation therapy; ΔBSI: BSI change on treatment; OS: Overall Survival; CSS: Cancer specific survival; PFS: Progression free survival; PSA-RS: Prostate specific antigen biochemical recurrence survival.

### Quality assessment

While there was small variation in the methodological quality of included studies, all 14 included studies were judged as moderate to relative high quality according to the Newcastle–Ottawa Quality Assessment Scale (NOS) assessment tool, with scores from 6 (5 studies) to 7 (9 studies, Supplementary Table 1).

### BSI and survival of mPCa

**Figure 2:**
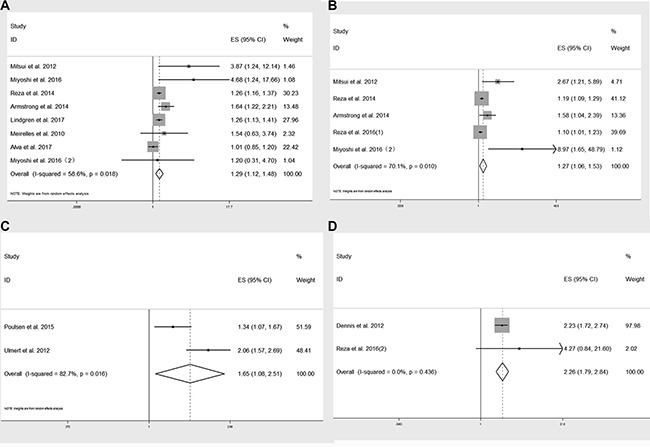
Forest plot of pooled HRs of baseline BSI in predicting OS (**A**), CSS (**C**), PSA response survival (**D**) and ΔBSI in predicting OS (**B**).

As displayed in Figure 2A, the forest plot showed high baseline BSI was significantly associated with poor overall survival (OS). The pooled HR was 1.29 (95% confidence interval [CI]: 1.12–1.48, *P* < 0.001) from 8 studies. Considering the high heterogeneity (*I^2^* = 58.6%, *P* = 0.018 ), we used random-effect model to pool the above variables.

Elevated BSI change on treatment (ΔBSI) was also correlated with poor OS, with a pooled HR of 1.27 (95% CI: 1.06–1.53, *P* < 0.001) from 5 studies (Figure 2B).

Furthermore, baseline BSI was also prominently related to cancer specific survival (CSS) and prostate specific antigen biochemical recurrence survival (PSA-RS). The pooled HR were 1.65 (95% CI: 1.08–2.51, *P* = 0.019, Figure 2C) and 2.26 (95% CI: 1.79–2.84, *P* < 0.001, Figure 2D), respectively.

**Figure 3:**
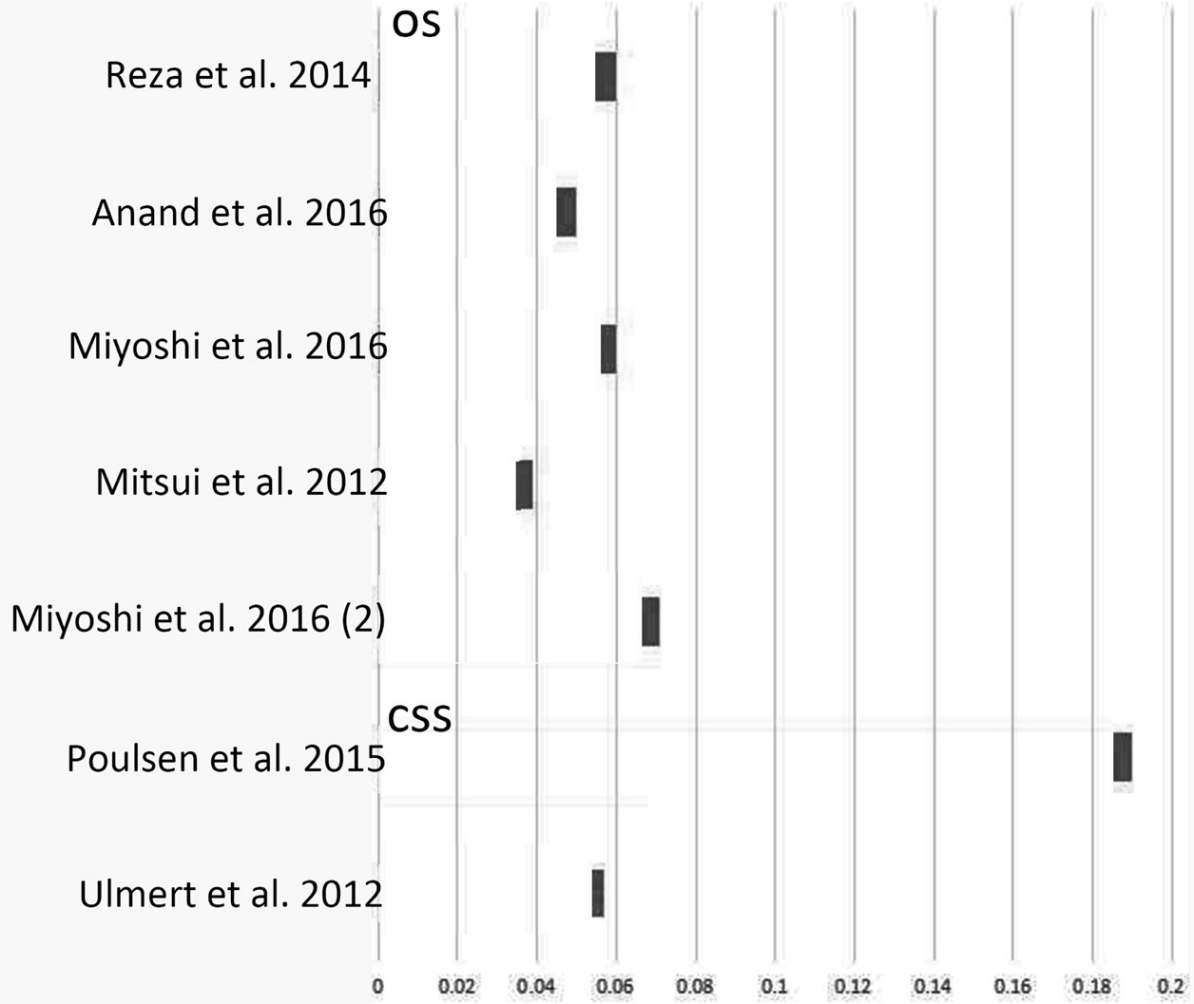
ΔC-index by adding BSI to baseline predicting models

There were 7 studies providing concordance index (C-index) of BSI on OS and 2 on CSS (Table 2). We calculated ΔC-index, which represented the improvement of efficacy by adding BSI into the baseline predicting models. As clearly showed in Figure 3, BSI could increase the predicting ability of OS and CSS in mPCa (all ΔC-indices were greater than zero).

**Table 2:** C-indices of predicting models with or without BSI

Study ID	C-index (95% CI) including BSI	Baseline c-index	ΔC-index	Survival outcome
Reza et al. 2014	0.83	0.77	0.06	OS
Anand et al. 2016	0.72	0.67	0.05	OS
Miyoshi et al. 2016	0.811	0.751	0.06	OS
Mitsui et al. 2012	0.66	0.621	0.039	OS
Miyoshi et al. 2016 (2)	0.792	0.721	0.071	OS
Lindgren et al. 2017	0.68	NR		OS
Reza et al. 2016 (1)	0.661	NR		OS
Poulsen et al. 2015	0.95 (0.81–1.0)	0.76 (0.39–1.0)	0.19	CSS
Ulmert et al. 2012	0.825 (0.754–0.881)	0.768 (0.702–0.837)	0.057	CSS

NR: not reported; OS: Overall Survival; CSS: Cancer specific survival.

### Subgroup analysis

**Figure 4:**
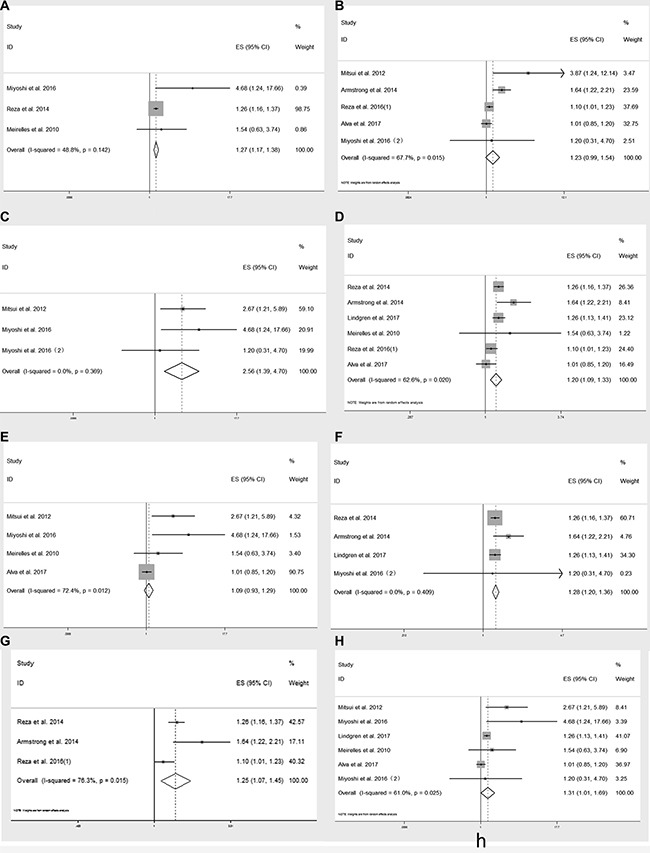
Subgroup analysis on baseline BSI and OS in patients with mPCa (**A**), mCRPC (**B**) ; Asians (**C**), non-Asians (**D**) ; cut-off value > 1 (**E**), cut-off value ≤ 1 (**F**) and sample size > 80 (**G**), sample size < 80 (**H**).

To deeply explore the relationship between BSI and OS, we performed subgroup analysis based on different types of mPCa, ethnics, cut-off values and sample sizes. The results were summarized in Table 3, with corresponding forest plots in Figure 4.

**Table 3:** Summary of the subgroup analysis results of BSI and OS

Variable	Number of studies	Number of patients	Model	Outcome (OS)		Heterogeneity	
				HR (95% CI)	*P* value	*I*-square (%)	*P* value
PCa type							
mPCa	3	224	F	1.269 (1.168–1.378)	< 0.001	48.8	0.142
mCRPC	5	336	R	1.230 (0.985–1.536)	0.067	67.7	0.015
Ethnicity							
Asian	3	142	F	2.558 (1.393–4.698)	0.002	0	0.369
Non-Asian	6	466	R	1.205 (1.090–1.332)	< 0.001	62.6	0.020
BSI cut-off							
> 1	4	210	R	1.094 (0.928–1.289)	0.285	72.4	0.012
≤ 1	4	294	F	1.276 (1.196–1.361)	< 0.001	0	0.409
Sample size							
> 80	3	310	R	1.248 (1.071–1.453)	0.004	76.3	0.015
< 80	6	298	R	1.309 (1.015–1.688)	0.038	61.0	0.025

F: fixed-effects model; R: random-effects model.

### Sensitivity analysis

**Figure 5:**
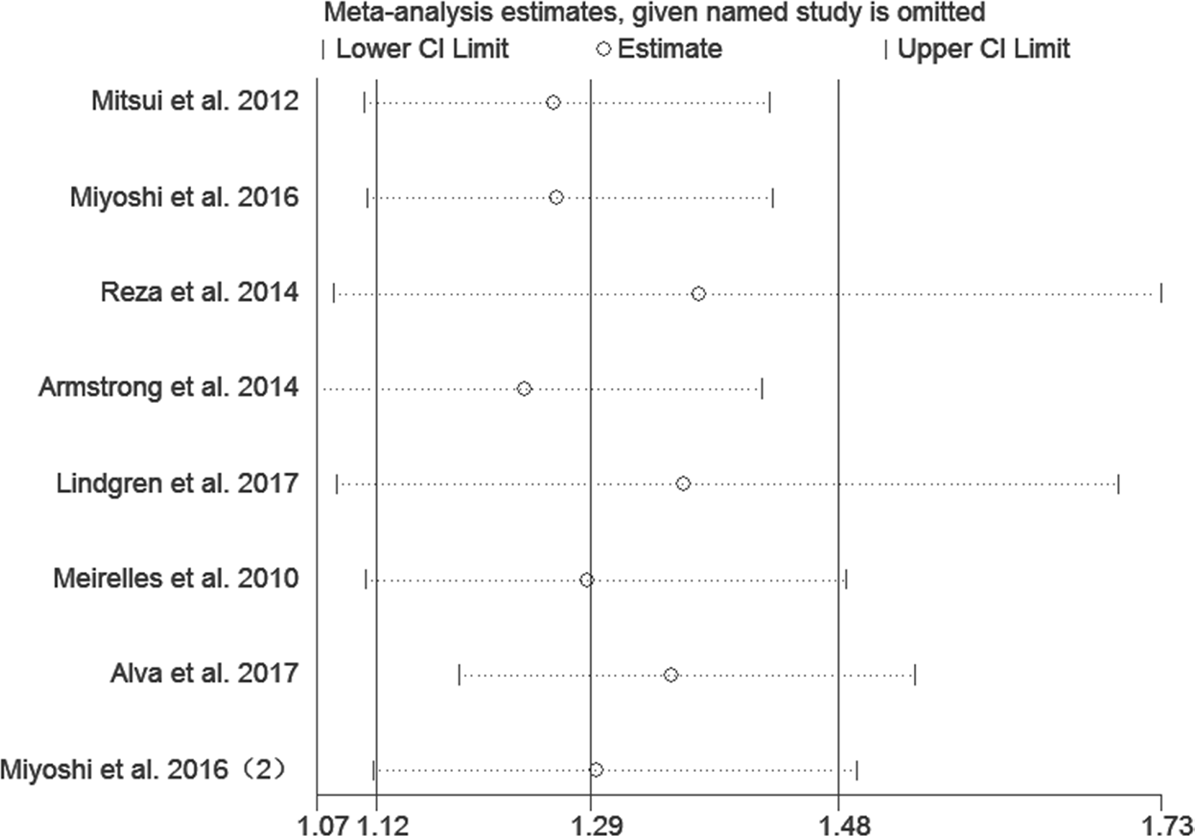
Sensitivity analysis of included studies

In order to gauge the stability of the results, we conducted sensitivity analysis by removing one study in sequence to see if a single study could have significant impact on the pooled HRs for OS. The results were not significantly altered by removing anyone of the included studies (Figure 5).

### Publication bias

**Figure 6:**
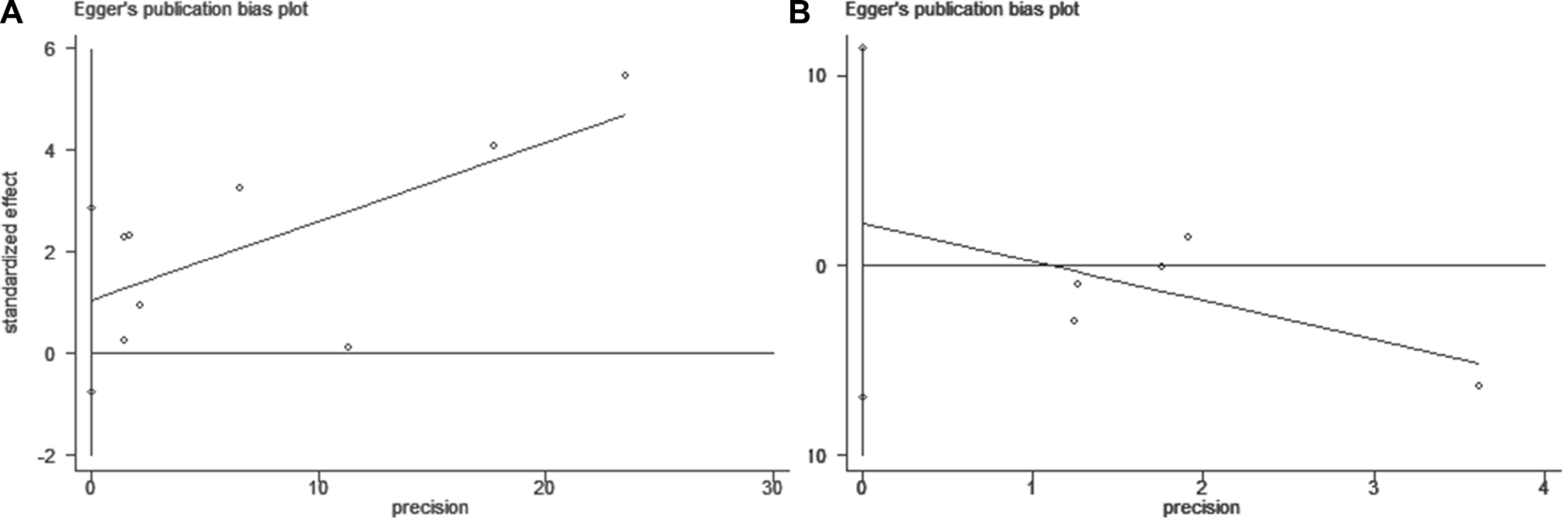
Funnel plot of Egger's test

The funnel plot revealed no significant publication bias in the meta-analysis of baseline BSI and OS (Figure 6A, Egger's test: *P* value = 0.2 ; Begg's test: *P* value = 0.386). Moreover, there was also no potential publication bias on ΔBSI and OS in patients with mPCa (Figure 6B, Egger's test: *P* value = 0.488 ; Begg's test: *P* value = 0.806).

## DISCUSSION

Currently, no standard quantitative imaging biomarkers are available to monitor the clinical changes during treatment in patients with mPCa. Positron emission tomography (PET) has higher sensitivity than plain film, but the results are also not quantitative. Besides, the PET's cost is significantly higher than bone scan’s, thus not easy to be popularized in the primary hospitals [22]. According to the Food and Drug Administration, a clinical validating biomarker should be measured reproducibly and consistently [23] , while automated BSI showed great potential in mPCa prognosis.

In this meta-analysis, based on the existing data from 14 included studies, the pooled results demonstrated that high baseline BSI indicated unfavorable poor OS, CSS and PSA-RS survival among mPCa patients. Elevated ΔBSI was significantly related to poor OS, which meant the potential feature on treatment monitoring. Given that BSI were acquired at different phases, we could tell if bone metastasis became progressive or stayed indolent, in that progression free survival (PFS) was predictive of OS in men with CRPC [24]. However, only 2 study [12, 21] explored the relationship between BSI and PFS, and the raw data was not sufficient to conduct meta-analysis on PFS.

Considering the clinical trails included which couldn't avoid heterogeneity since they enrolled in mPCa patients with different stages and involved various treatments. We then performed subgroup analysis. BSI showed predictive value in mPCa patients who mainly received androgen deprivation therapy (ADT, HR = 1.269, 95% CI: 1.168–1.378), however, BSI was not significantly associated with OS in mCRPC (HR = 1.230, 95% CI: 0.985–1.536). Among mCRPC group, the treatment still varied from chemotherapy, tasquinimod, enzalutamide to radium-223. Since the lack of studies, we couldn't conduct subgroup analysis on each treatment, so this result should be cautiously interpreted. Ethnicity might serve as a major source of heterogeneity, then we found BSI was significantly correlated with poor OS in Asian patients (HR = 2.558, 95% CI: 1.393–4.698, *I^2^* = 0) and non-Asian group (HR = 1.205, 95% CI: 1.090–1.332, *I^2^* = 62.6%). Because the 3 cohorts from Asian used BONENAVI system which were adjusted by large clinical data from Japanese patients [25], there was no obvious heterogeneity. On the contrary, the non-Asian group contained patients from Europe and USA, who were mainly Caucasians with a small portion of African-American, therefore, which might contribute to heterogeneity. From another aspect, our result supported the main result when cut-off value ≤ 1 (HR = 1.276, 95% CI: 1.196–1.361, *I^2^* = 0), since most studies used the 1 as BSI cut-off [10, 12, 13, 18, 19]. What's more, the result wasn't affected by different sample sizes.

To test the performance of a prognostic model, C-index was known as a parameter like area under the summary receiver operating characteristic curve (SROC) [26]. The range of C-index was 0.5–1.0, and higher C-index meant better efficacy of predicting model. In our study, ΔC-indices were calculated and displayed in a graph, which had clearly shown the added value of BSI to traditional models including clinical T stage, PSA and Gleason score.

Though we failed to conduct a meta-analysis on the relation (r) between BSI and PSA owing to insufficient information, BSI was testified correlated with serum PSA change in several studies [8, 11, 13, 20], using different methods such as Kendall's tau, Pearson and Spearman tests. However, Poulsen et al. [27] reported that only BSI served as an independent prognostic factor for survival of men with mPCa, PSA and Gleason scores were not.

To the best of our knowledge, this is the first meta-analysis about BSI and prognosis in mPCa. However, there are still several limitations in the present study. First, among the 14 included studies, only 2 studies were separately eligible for CSS or PSA-RS and 7 studies available for ΔC-index analysis. The number of studies was relatively small. Second, although sensitivity analysis supported the stability of our results, the findings should be cautiously interpreted. Heterogeneity among studies was found probably because of relatively small sample sizes and multivariate influence factors in some included studies. Third, we lack the BSI data and other corresponding clinical parameters on different population at present. Large scale statistics about BSI response during different treatment such as chemotherapy and radiotherapy are also insufficient. So further prospective clinical trials with large sample size are required to verify the prognostic value of BSI on mPCa patients in the future.

## MATERIALS AND METHODS

### Search strategy

This meta-analysis was conducted under the guidelines of the Preferred Reporting Items for Systematic Reviews and Meta-Analyses (PRISMA) [28]. A comprehensive literature search for relevant studies in the PubMed, Embase, Web of Science and the Cochrane library was performed through May 19, 2017. The searching strategy consisted of medical subheadings and key words. The main terms were as follows: ’prostate neoplasms [MeSH]’ or ’castration resistant prostate cancer’ or ‘bone metastasis’ or ’metastatic prostate cancer’ and ’bone scan index’ or ’BSI’ and ’prognosis [MeSH]’ or ’survival’ or ’outcome’. The language of studies, population and sample size were not restricted. We also manually searched the reference lists for additional relevant publications.

## STUDY SELECTION

### Inclusion and exclusion criteria

Studies meeting the following criteria were considered eligible: 1. clinical cohort evaluated the prognostic accuracy of BSI in mPCa; 2. studies compared BSI with other prognosis models and reported survival outcomes like OS, CSS, PFS ; 3. reported original C-index or HR with 95%CI or HR could be extracted from sufficient information; 4. articles with the most complete information if there were multiple studies on the same cohort.

The exclusion criteria were: 1. repeated publications; 2. studies reporting on less than 20 patients; 3. experimental laboratory articles, animal studies, letters or review articles.

### Assessment of study quality

Two investigators (D.L. and H.L.) independently reviewed all relevant articles, then judged the methodology quality of potential studies using NOS assessment tool, including selection, comparability and outcome [29]. A study was considered high quality if the NOS score ≥ 7. When disagreements occurred, the two reviewers reached consensus by involving a third author (H.D.).

### Data extraction

We extracted the following variables from each study: first author's name; publication year; study design; country or region of the study; BSI software type (manufacturer); sample size; age, PSA, Gleason score; cut-off value; follow up time, out-come assessment and risk estimates, C-indices and HRs with 95% CI. If the HRs of both univariate and multivariate analysis for the same comparison were available, we only used the latter. If the HR and 95% CI were not displayed directly, they were estimated from Kaplan–Meier curves [30]. If necessary, the corresponding author could be contacted for further information.

### Statistical analysis

HRs with 95%CI were pooled using a meta-analysis to access the strength of BSI to survival endpoints. C-indices of baseline models and the models adding BSI were extracted and the difference values, ΔC-index, were calculated. The Cochrane Q test was used to determine the heterogeneity among studies. A *P* value < 0.10 indicated heterogeneity. I-square (*I^2^*) was also calculated to evaluate heterogeneity. An *I^2^* value > 50% was considered significant heterogeneity. The fixed-effect model was used to calculate pooled results when no heterogeneity existed among included studies, otherwise, a random-effect model was used. To find reasons of heterogeneity among studies, we conducted subgroup analysis in different types of mPCa, ethnics, cut-off values and sample sizes, respectively. To test the reliability of the main outcomes in our analysis, sensitivity analysis was performed by removing one single study in turn. Egger's and Begg's tests with funnel plots were used to test publication bias. *P* value > 0.05 indicated no potential publication bias. Kaplan–Meier curves were read by Engauge Digitizer version 9.8 (http://markummitchell.github.io/engauge-digitizer/). We used Stata 12.0 software (Stata Corporation, College Station, TX, USA) to conduct all the statistical analyses. A two-sided *P* value less than 0.05 was considered statistically significant.

## CONCLUSIONS

Our study demonstrates that BSI may be beneficial as a predictive imaging marker in mPCa prognosis. We deem that ,with the high prognostic value, baseline BSI and ΔBSI may contribute to monitor and treatment in patients with mPCa.

## SUPPLEMENTARY MATERIALS TABLES





## Abbreviations

AA: abiraterone acetate
ADT: androgen deprivation therapy
BSI: bone scan index
CI: confidence interval
C-index: concordance index
CRPC: castration-resistant prostate cancer
CSS: cancer specific survival
ENZ: Enzalutamide
HRs: hazard ratios
mPCa: metastatic prostate cancer
mCRPC: metastatic castration-resistant prostate cancer
NOS: Newcastle–Ottawa Quality Assessment Scale
NR: not reported
OS: overall survival
PCa: prostate cancer
PET: positron emission tomography
PFS: progression free survival
PSA: prostate specific antigen
PSA-RS: prostate specific antigen biochemical recurrence survival
PRISMA: Preferred Reporting Items for Systemic Reviews and Meta-Analyses
SROC: summary receiver operating characteristic curve
ΔBSI: BSI change on treatment

## References

[1] Siegel RL, Miller KD, Jemal A (2017). Cancer Statistics, 2017. CA Cancer J Clin.

[2] Groot MT, Boeken Kruger CG, Pelger RC, Uyl-de Groot CA (2003). Costs of prostate cancer, metastatic to the bone, in the Netherlands. Eur Urol.

[3] Heidenreich A, Bastian PJ, Bellmunt J, Bolla M, Joniau S, van der Kwast T, Mason M, Matveev V, Wiegel T, Zattoni F, Mottet N (2014). EAU guidelines on prostate cancer. Part II: Treatment of advanced, relapsing, and castration-resistant prostate cancer. Eur Urol.

[4] Bitting RL, Armstrong AJ (2013). Potential predictive biomarkers for individualizing treatment for men with castration-resistant prostate cancer. Cancer J.

[5] Imbriaco M, Larson SM, Yeung HW, Mawlawi OR, Erdi Y, Venkatraman ES, Scher HI (1998). A new parameter for measuring metastatic bone involvement by prostate cancer: the Bone Scan Index. Clin Cancer Res.

[6] Ulmert D, Kaboteh R, Fox JJ, Savage C, Evans MJ, Lilja H, Abrahamsson PA, Bjork T, Gerdtsson A, Bjartell A, Gjertsson P, Hoglund P, Lomsky M (2012). A novel automated platform for quantifying the extent of skeletal tumour involvement in prostate cancer patients using the Bone Scan Index. Eur Urol.

[7] Kaboteh R, Gjertsson P, Leek H, Lomsky M, Ohlsson M, Sjostrand K, Edenbrandt L (2013). Progression of bone metastases in patients with prostate cancer - automated detection of new lesions and calculation of bone scan index. EJNMMI Res.

[8] Mitsui Y, Shiina H, Yamamoto Y, Haramoto M, Arichi N, Yasumoto H, Kitagaki H, Igawa M (2012). Prediction of survival benefit using an automated bone scan index in patients with castration-resistant prostate cancer. BJU Int.

[9] Miyoshi Y, Yoneyama S, Kawahara T, Hattori Y, Teranishi J, Kondo K, Moriyama M, Takebayashi S, Yokomizo Y, Yao M, Uemura H, Noguchi K (2016). Prognostic value of the bone scan index using a computer-aided diagnosis system for bone scans in hormone-naive prostate cancer patients with bone metastases. BMC Cancer.

[10] Miyoshi Y, Uemura K, Kawahara T, Yoneyama S, Hattori Y, Teranishi JI, Ohta JI, Takebayashi S, Yokomizo Y, Hayashi N, Yao M, Uemura H (2016). Prognostic Value of Automated Bone Scan Index in Men With Metastatic Castration-resistant Prostate Cancer Treated With Enzalutamide or Abiraterone Acetate. Clin Genitourin Cancer.

[11] Anand A, Morris MJ, Larson SM, Minarik D, Josefsson A, Helgstrand JT, Oturai PS, Edenbrandt L, Roder MA, Bjartell A (2016). Automated Bone Scan Index as a quantitative imaging biomarker in metastatic castration-resistant prostate cancer patients being treated with enzalutamide. EJNMMI Res.

[12] Reza M, Bjartell A, Ohlsson M, Kaboteh R, Wollmer P, Edenbrandt L, Tragardh E (2014). Bone Scan Index as a prognostic imaging biomarker during androgen deprivation therapy. EJNMMI Res.

[13] Armstrong AJ, Kaboteh R, Carducci MA, Damber JE, Stadler WM, Hansen M, Edenbrandt L, Forsberg G, Nordle O, Pili R, Morris MJ (2014). Assessment of the bone scan index in a randomized placebo-controlled trial of tasquinimod in men with metastatic castration-resistant prostate cancer (mCRPC). Urol Oncol.

[14] Lindgren Belal S, Sadik M, Kaboteh R, Hasani N, Enqvist O, Svarm L, Kahl F, Simonsen J, Poulsen MH, Ohlsson M, Hoilund-Carlsen PF, Edenbrandt L, Tragardh E (2017). 3D skeletal uptake of 18F sodium fluoride in PET/CT images is associated with overall survival in patients with prostate cancer. EJNMMI Res.

[15] Meirelles GS, Schoder H, Ravizzini GC, Gonen M, Fox JJ, Humm J, Morris MJ, Scher HI, Larson SM (2010). Prognostic value of baseline [18F] fluorodeoxyglucose positron emission tomography and 99mTc-MDP bone scan in progressing metastatic prostate cancer. Clin Cancer Res.

[16] Reza M, Ohlsson M, Kaboteh R, Anand A, Franck-Lissbrant I, Damber JE, Widmark A, Thellenberg-Karlsson C, Budaus L, Steuber T, Eichenauer T, Wollmer P, Edenbrandt L (2016). Bone Scan Index as an Imaging Biomarker in Metastatic Castration-resistant Prostate Cancer: A Multicentre Study Based on Patients Treated with Abiraterone Acetate (Zytiga) in Clinical Practice. Eur Urol Focus.

[17] Alva A, Nordquist L, Daignault S, George S, Ramos J, Albany C, Isharwal S, McDonald M, Campbell G, Danchaivijitr P, Yentz S, Anand A, Yu EY (2017). Clinical Correlates of Benefit From Radium-223 Therapy in Metastatic Castration Resistant Prostate Cancer. Prostate.

[18] Poulsen MH, Rasmussen J, Edenbrandt L, Hoilund-Carlsen PF, Gerke O, Johansen A, Lund L (2016). Bone Scan Index predicts outcome in patients with metastatic hormone-sensitive prostate cancer. BJU Int.

[19] Ulmert D, Kaboteh R, Fox JJ, Savage C, Evans MJ, Lilja H, Abrahamsson PA, Bjork T, Gerdtsson A, Bjartell A, Gjertsson P, Hoglund P, Lomsky M (2012). A novel automated platform for quantifying the extent of skeletal tumour involvement in prostate cancer patients using the Bone Scan Index. Eur Urol.

[20] Dennis ER, Jia X, Mezheritskiy IS, Stephenson RD, Schoder H, Fox JJ, Heller G, Scher HI, Larson SM, Morris MJ (2012). Bone scan index: a quantitative treatment response biomarker for castration-resistant metastatic prostate cancer. J Clin Oncol.

[21] Reza M, Jones R, Aspegren J, Massard C, Mattila L, Mustonen M, Wollmer P, Tragardh E, Bondesson E, Edenbrandt L, Fizazi K, Bone Bjartell A (2016). Scan Index and Progression-free Survival Data for Progressive Metastatic Castration-resistant Prostate Cancer Patients Who Received ODM-201 in the ARADES Multicentre Study. Eur Urol Focus.

[22] Pinto F, Totaro A, Palermo G, Calarco A, Sacco E, D'Addessi A, Racioppi M, Valentini A, Gui B, Bassi P (2012). Imaging in prostate cancer staging: present role and future perspectives. Urol Int.

[23] Goodsaid F, Frueh F (2007). Biomarker qualification pilot process at the US Food and Drug Administration. Aaps j.

[24] Halabi S, Vogelzang NJ, Ou SS, Owzar K, Archer L, Small EJ (2009). Progression-free survival as a predictor of overall survival in men with castrate-resistant prostate cancer. J Clin Oncol.

[25] Koizumi M, Wagatsuma K, Miyaji N, Murata T, Miwa K, Takiguchi T, Makino T, Koyama M (2015). Evaluation of a computer-assisted diagnosis system, BONENAVI version 2, for bone scintigraphy in cancer patients in a routine clinical setting. Ann Nucl Med.

[26] Uno H, Cai T, Pencina MJ, D'Agostino RB, Wei LJ (2011). On the C-statistics for evaluating overall adequacy of risk prediction procedures with censored survival data. Stat Med.

[27] Poulsen MH, Rasmussen J, Edenbrandt L, Hoilund-Carlsen PF, Gerke O, Johansen A, Lund L (2016). Bone Scan Index predicts outcome in patients with metastatic hormone-sensitive prostate cancer. BJU Int.

[28] Moher D, Liberati A, Tetzlaff J, Altman DG (2010). Preferred reporting items for systematic reviews and meta-analyses: the PRISMA statement. Int J Surg.

[29] Stang A (2010). Critical evaluation of the Newcastle-Ottawa scale for the assessment of the quality of nonrandomized studies in meta-analyses. Eur J Epidemiol.

[30] Tierney JF, Stewart LA, Ghersi D, Burdett S, Sydes MR (2007). Practical methods for incorporating summary time-to-event data into meta-analysis. Trials.

