# Supplementary material for: Prognostic value of bone scan index as an imaging biomarker in metastatic prostate cancer: a meta-analysis
Source: Oncotarget. 2017 Jul 29;8(48):84449–58. doi: 10.18632/oncotarget.19680 (PMC5663610; doi:10.18632/oncotarget.19680)
Supplement: Supplementary file 1 [file oncotarget-08-84449-s001.pdf]

Prognostic value of bone scan index as an imaging biomarker in metastatic prostate cancer: a meta-analysis

SUPPLEMENTARY MATERIALS

Supplementary Table 1: NOS scores of included studies. See Supplementary_Table_1
